# Antioxidant Peptides from the Protein Hydrolysate of Monkfish (*Lophius litulon*) Muscle: Purification, Identification, and Cytoprotective Function on HepG2 Cells Damage by H_2_O_2_

**DOI:** 10.3390/md18030153

**Published:** 2020-03-10

**Authors:** Xiao-Meng Hu, Yu-Mei Wang, Yu-Qin Zhao, Chang-Feng Chi, Bin Wang

**Affiliations:** 1 Zhejiang Provincial Engineering Technology Research Center of Marine Biomedical Products, School of Food and Pharmacy, Zhejiang Ocean University, Zhoushan 316022, China; hu970203@163.com (X.-M.H.); wangym731@126.com (Y.-M.W.); zhaoy@hotmail.com (Y.-Q.Z.); 2 National and Provincial Joint Laboratory of Exploration and Utilization of Marine Aquatic Genetic Resources, National Engineering Research Center of Marine Facilities Aquaculture, School of Marine Science and Technology, Zhejiang Ocean University, Zhoushan 316022, China

**Keywords:** monkfish (*Lophius litulon*), muscle, antioxidant peptide, HepG2 cell, cytoprotective activity

## Abstract

In the work, defatted muscle proteins of monkfish (*Lophius litulon*) were separately hydrolyzed by pepsin, trypsin, and in vitro gastrointestinal (GI) digestion methods, and antioxidant peptides were isolated from proteins hydrolysate of monkfish muscle using ultrafiltration and chromatography processes. The antioxidant activities of isolated peptides were evaluated using radical scavenging and lipid peroxidation assays and H_2_O_2_-induced model of HepG2 cells. In which, the cell viability, reactive oxygen species (ROS) content, and antioxidant enzymes and malondialdehyde (MDA) levels were measured for evaluating the protective extent on HepG2 cells damaged by H_2_O_2_. The results indicated that the hydrolysate (MPTH) prepared using in vitro GI digestion method showed the highest degree of hydrolysis (27.24 ± 1.57%) and scavenging activity on a 2,2-diphenyl-1-picrylhydrazyl (DPPH) radical (44.54 ± 3.12%) and hydroxyl radical (41.32 ± 2.73%) at the concentration of 5 mg protein/mL among the three hydrolysates. Subsequently, thirteen antioxidant peptides (MMP-1 to MMP-13) were isolated from MPTH. According to their DPPH radical and hydroxyl radical scavenging activity, three peptides with the highest antioxidant activity were selected and identified as EDIVCW (MMP-4), MEPVW (MMP-7), and YWDAW (MMP-12) with molecular weights of 763.82, 660.75, and 739.75 Da, respectively. EDIVCW, MEPVW, and YWDAW showed high scavenging activities on DPPH radical (EC_50_ 0.39, 0.62, and 0.51 mg/mL, respectively), hydroxyl radical (EC_50_ 0.61, 0.38, and 0.32 mg/mL, respectively), and superoxide anion radical (EC_50_ 0.76, 0.94, 0.48 mg/mL, respectively). EDIVCW and YWDAW showed equivalent inhibiting ability on lipid peroxidation with glutathione in the linoleic acid model system. Moreover, EDIVCW, MEPVW, and YWDAW had no cytotoxicity to HepG2 cells at the concentration of 100.0 µM and could concentration-dependently protect HepG2 cells from H_2_O_2_-induced oxidative damage through decreasing the levels of reactive oxygen species (ROS) and MDA and activating intracellular antioxidant enzymes of superoxide dismutase (SOD), catalase (CAT), and glutathione peroxidase (GSH-Px). These present results indicated that the protein hydrolysate and isolated antioxidant peptides from monkfish muscle, especially YWDAW could serve as powerful antioxidants applied in the treatment of some liver diseases and healthcare products associated with oxidative stress.

## 1. Introduction

Reactive oxygen species (ROS) are generated as part of the normal metabolic processes and eliminated through enzymatic and non-enzymatic antioxidant defense systems in aerobic organisms [1,2]. However, the metabolic oxidant/antioxidant equilibrium is broken when the organisms are exposed to abnormal environments, including air pollutants, X-rays radiation, cigarette smoking, and industrial chemicals, which stimulate oxidative stress and generate superfluous and toxic ROS [3,4]. Those ROS lead to the development of aging and many chronic diseases, such as cardiovascular disease (CVD), inflammation, diabetes mellitus, neurodegenerative disorders, and cancers [5,6]. In addition, lipid corruption induced by oxidation in food is the main reason for the quality deterioration and shortening the shelf life [7,8,9]. At present, artificial antioxidants are generally applied to clear ROS in food and biological systems, but increasing evidence linking artificial antioxidants to negative health influences severely limited their applications [1,10]. Then, natural compounds, such as peptides, phenols, flavonoids, carotenoids, and vitamins, are continually investigated as the alternative agents of artificial antioxidants due to their significant antioxidant activities and little adverse effects [11].

Antioxidant peptides (APs) isolated from food proteins have drawn great public attention because of their high-level safety and strong ROS scavenging, cytoprotective, and DNA protection capacities [12,13,14]. EVSGPGLSPN from walnut could significantly (*p* < 0.05) reduce ROS generation, and dose-dependently increase cell viability of H_2_O_2_-induced PC12 cells by enhancing the levels of intracellular antioxidant enzymes and blocking the nuclear factor-κB (NF-κB)/caspase pathway [15]. IYVVDLR and IYVFVR from alcalase-hydrolysate of soybean could strongly scavenge radicals, upregulate total reduced glutathione (GSH) synthesis, enhance activities of intracellular antioxidant enzyme (catalase (CAT) and glutathione reductase (GR), and suppress ROS-mediated inflammatory responses via inhibiting interleukin-8 secretion in Caco-2 cells [16]. APs from hazelnut by-products [17] and corn gluten [18] exhibited similar cytoprotective effects and intracellular ROS scavenging activities through the upregulation of intracellular antioxidant enzyme and GSH levels.

In addition, APs from seafood and their processing by-products such as tuna, cuttlefish, sardinelle, bluefin leatherjacket, tilapia, and shrimp, also have been studied and showed potent antioxidant activities including radical scavenging activity, reducing power, and inhibition ability on lipid and protein oxidation [5,19]. Zhao et al. reported that FPYLRH and GIEWA from protein hydrolysate of miiuy croaker swim bladders could strongly scavenge radicals and effectively inhibit lipid peroxidation [20]. PIIVYWK and FSVVPSPK from *Mytilus edulis* hydrolysates exhibited strong 2,2-diphenyl-1-picrylhydrazyl radical (DPPH·) scavenging and ORAC activities, and increased cell viabilities in H_2_O_2_-induced hepatotoxicity through upregulating heme oxygenase-1 (HO-1) level under normal and oxidative stress conditions in cultured hepatocytes [21]. WAFAPA and MYPGLA from hydrolysate of blue-spotted stingray surpassed carnosine in their abilities to suppress H_2_O_2_-induced lipid oxidation and protected plasmid DNA and proteins from Fenton’s reagent-induced oxidative damage [22]. Therefore, marine proteins are high-quality raw materials for the preparation of APs with the ability to enhance health by reducing oxidative stress. Monkfish (*Lophius litulon*) belongs to the family of Lophiidae and is a common commercial fish with the production of approximately 2 × 10^5^ tons/year [7]. Therefore, the hydrolysis method of monkfish (*Lophius litulon*) muscle proteins was optimized and three novel APs with high radical scavenging and cytoprotective activities were isolated and identified from the hydrolysate prepared using in vitro GI digestion method. In addition, the effects of three isolated APs on cell viability, ROS content, and antioxidant enzymes and malondialdehyde (MDA) levels in H_2_O_2_-induced model of HepG2 cells were measured. These results would provide a better understanding of the exploitation of the marine resource for bioactive peptides and lay the groundwork necessary for elucidating the antioxidant mechanism and development of functional foods using antioxidant hydrolysate and peptides of monkfish.

## 2. Materials and Methods

### 2.1. Materials

Monkfish (*L. litulon*) was purchased from Fengmao Market in Zhoushan city of China. DEAE-52 cellulose and Sephadex G-15 were purchased from Shanghai Source Poly Biological Technology Co., Ltd. (Shanghai, China). Acetonitrile (ACN) of LC grade and trifluoroacetic acid (TFA) were purchased from Thermo Fisher Scientific Co., Ltd. (Shanghai, China). HepG2 cells were purchased from the Cell Bank of Type Culture Collection of the Chinese Academy of Sciences (Shanghai, China). Phosphate buffered saline (PBS, pH 7.2), dimethyl sulfoxide (DMSO), Dulbecco’s modified Eagle’s medium (DMEM), 3-(4, 5-dimethylthiazol-2-y1)-2,5-diphenyltetrazo lium bromide (MTT), L-glutamine, and DPPH were purchased from Sigma-Aldrich (Shanghai) (Trading Co., Ltd. Shanghai, China). EDIVCW (MMP-4), MEPVW (MMP-7), and YWDAW (MMP-12) with purity higher than 98% were synthesized in China Peptides Co. Ltd. (Suzhou, China).

### 2.2. Preparation of Protein Hydrolysate from Monkfish Muscle 

The monkfish muscle was defatted according to our previous method [7]. Afterwards, the defatted muscle was hydrolyzed by pepsin, trypsin and in vitro GI digestion (pepsin–trypsin system) [2,9,23]. The hydrolytic process of the defatted monkfish muscles separately using pepsin and trypsin was performed following the previous methods [9]. The dispersions of the defatted muscle (1%, w/v) were hydrolyzed separately on their optimal hydrolysis parameters (pepsin at pH 2.0, 37 °C with total enzyme dose 2.0%; trypsin at pH 8.0, 40 °C with a total enzyme dose of 2.0%).

The protein hydrolysate of monkfish muscles prepared using the in vitro GI digestion was performed according to the previous method [23]. The defatted muscle dispersions (pH 1.5, 1%, w/v) were hydrolyzed using pepsin at 37.0 °C with a total enzyme dose of 1% (w/w). In 2 h, the pH value of the mixture was adjusted to 7.0 with NaOH solution (1.0 M) and hydrolyzed using trypsin at 37.0 °C with a total enzyme dose of 1% (w/w) for 2 h. After that, the hydrolysate was kept in a 95 °C water bath for 10 min and centrifuged at 9000× *g* for 20 min. The resulting supernatants were freeze-dried and store in a −20 °C refrigerator. The protein hydrolysate of monkfish muscles prepared using the in vitro GI digestion method was referred to as hydrolysate (MPTH). The concentrations of protein hydrolysates and their fractions were expressed as mg protein/mL and measured by the dye-binding method of Bradford [24]. The degree of hydrolysis (DH, %) of hydrolysates was measured according to the previous method [25].

### 2.3. Isolation of APs from MPTH

The isolation process of APs from MPTH was performed according to the previous method [26]. MPTH was fractionated using 3 and 5 kDa molecular weight (MW) cut-off membranes, and three fractions termed MPTH-I (MW < 3 kDa), MPTH-II (3 kDa < MW < 5 kDa), and MPTH-III (MW > 5 kDa) were collected and lyophilized. MPTH-I solution (5 mL, 40.0 mg protein/mL) was injected into a DEAE-52 cellulose column (1.6 × 80 cm) pre-equilibrated with deionized water (DW), and stepwise eluted with 150 mL of DW and NaCl solution (0.1, 0.5, and 1.0 M, respectively) at a flow rate of 1.0 mL/min, respectively. Each eluate (5 mL) was monitored at 214 nm. Six fractions (MUA-1 to MUA-6) were pooled on the chromatographic peaks and MUA-5 solution (5 mL, 10.0 mg protein/mL) was separated on a Sephadex G-25 column (2.6 × 160 cm) eluted with DW at a flow rate of 0.6 mL/min. Each eluate (3 mL) was collected, monitored at 214 nm, and measured the DPPH· and hydroxyl radical (HO·) scavenging activity. Two subfractions (MUA-4-A and MUA-4-B) were collected and MUA-4-B with higher DPPH· and HO· scavenging activity was isolated using a Zorbax, SB C-18 column (4.6 × 250 mm, 5 µm) in an Agilent 1260 HPLC system. The sample was eluted with a linear gradient of acetonitrile (0%–40% in 0–40 min) in 0.1% TFA at a flow rate of 1.0 mL/min. Thirteen APs (MMP-1 to MMP-13) were isolated on the absorbance at 214 nm and lyophilized.

### 2.4. Analysis of Amino Acid Sequence and MW

The amino acid sequences and molecular masses of three isolated peptides (MMP-4, MMP-7, and MMP-12) were measured on an Applied Biosystems 494 protein sequencer (Perkin Elmer/Applied Biosystems Inc., Foster City, CA, USA) and a quadrupole time-of-flight mass spectrometer (Q-TOF MS) coupled with an electrospray ionization source (ESI) (Micromass, Waters, Los Angeles, CA, USA), respectively.

### 2.5. Radical Scavenging and Lipid Peroxidation Inhibition Assays

The DPPH·, HO·, and superoxide anion radical ($O_{2}^{-}$·) scavenging activities assays were measured on the previous methods [7,9]. The half-elimination ratio (EC_50_) was defined as the concentration where a sample caused a 50% decrease in the initial concentration of DPPH·, HO·, and $O_{2}^{-}$·, respectively.

### 2.6. Lipid Peroxidation Inhibition Assay

The lipid peroxidation inhibition activity of the APs was measured in a linoleic acid model system using the method of Wang et al. [7,9]. Briefly, a sample (5.0 mg) was dissolved in 10 mL of 50 mM PBS (pH 7.0) and added to 0.13 mL of a solution of linoleic acid and 10 mL of 99.5% ethanol. Then, the total volume was adjusted to 25 mL with deionized water. The mixture was incubated in a conical flask with a screw cap at 40 °C in a dark room, and the degree of oxidation was evaluated by measuring ferric thiocyanate values. The reaction solution (100 μL) incubated in the linoleic acid model system was mixed with 4.7 mL of 75% ethanol, 0.1 mL of 30% ammonium thiocyanate, and 0.1 mL of 20 mM ferrous chloride solution in 3.5% HCl. After 3 min, the thiocyanate value was measured at 500 nm following color development with FeCl_2_ and thiocyanate at different intervals during the incubation period at 40 °C.

### 2.7. Cell Culture and Cytotoxicity Assay 

MTT test was performed on the previous method to measure the Cytotoxicity of samples [2]. In brief, the HepG2 cells were cultured in DMEM medium containing 10% FBS supplemented with 2 mM L-Glu, and 1% penicillin–streptomycin solution at 37 °C and 5% CO_2_ atmosphere. After 24 h incubation in a 96-well plate (7 × 10 ^3^ cells/well), the HepG2 cells were cultured at the presence of designed concentrations of peptide solution for 12 h. After that, the wells were washed with PBS twice and the MTT with the final concentration of 0.5 mg/mL was added into. After 4 h, the formazan crystals formed by active cells were dissolved in 150 µL of DMSO and the absorbance at 570 nm of the solution was recorded. The cell viability was calculated by the following equation:

Cell viability = (A_sample_/A_control_) × 100%. (1)

### 2.8. The Cytoprotective Activity of APs on Oxidative Damaged HepG2 Cells by H_2_O_2_

The assay was performed on the previous method and the H_2_O_2_ concentration of 300 µM was used to build the oxidative damage model of HepG2 cells [2]. In brief, the isolated peptides (EDIVCW (MMP-4), MEPVW (MMP-7), and YWDAW (MMP-12)) were dissolved in the DMEM medium with the concentrations of 10.0, 50.0, and 100.0 µM. The HepG2 cells were grown (6 × 10^4^ cells/well) in a 96-well plate for 24 h. Then the supernatant was aspirated and 100 µL of peptide samples were added into the protection groups respectively for incubating 8 h. After removing peptides from sample groups, H_2_O_2_ was respectively added into the damage and protection groups with the optimal concentration of 300 µM and the mixed solutions were subsequently incubated for 24 h. Finally, the cell viability was measured and calculated according to the method of Section 2.6. 

### 2.9. Determination of the Levels of ROS in H_2_O_2_-Induced HepG2 Cells

Intracellular ROS accumulation in HepG2 cells was monitored according to the previous method described by Zheng et al. [13]. In brief, HepG2 cells were preincubated with samples at the concentrations of 10, 50, or 100 μM for 12.0 h, and then incubated with H_2_O_2_ at the concentration of 300 µM for 2 h. After that, the cells were washed with PBS and incubated with 10 μM DCFH2-DA in fresh culture medium for 0.5 h. Intracellular ROS levels indicated by DCF fluorescence were quantified on a BD FACS Calibur flow cytometer (BD Biosciences, San Diego, C, USA) using excitation and emission filters of 488 and 530 nm, respectively. The data were expressed as % of control values.

### 2.10. Determination of the Levels of Antioxidant Enzymes and MDA in H_2_O_2_-Induced HepG2 Cells

The assay was performed according to the previous methods [2,27]. The HepG2 cells were cultured in 6-well plates (1 × 10^6^ cells/well). The isolated peptides (final concentration of 10.0, 50.0, and 100.0 µM, respectively) were added into the protection groups. After that, the damage and protection groups were induced by H_2_O_2_ at a concentration of 300 µM. Finally, 500 mL of cell lysis buffer was added into each well on ice lysed for 0.5 h and centrifuged at 12,000 g, 4 °C for 10 min. The resulted liquid supernatant was followed with cold standby at 4 °C (the indicators should be measured in 6 h). The levels of superoxide dismutase (SOD), CAT, GSH-Px and MDA were measured using assay kits according to the protocols of the manufacturer. The results were expressed as units of enzymatic activity per milligram of protein (U/mg prot).

### 2.11. Statistical Analysis

The data are expressed as the mean ± standard deviation (SD, *n* = 3). A one-way analysis of variance test for differences between means of each group was applied to analyze data using SPSS 19.0 (SPSS Corporation, Chicago, IL, USA), and a *p*-value (< 0.05) was considered statistically significant.

## 3. Results and Discussion

### 3.1. Preparation of the Protein Hydrolysate of Monkfish Muscle

The specificity of the enzyme significantly influences the bioactivity and production of APs [11]. In vitro GI digestion model is widely applied to investigate the digestibility, release of food components, and structural changes under simulated GI conditions [28]. Moreover, the model is popularly used to produce protein hydrolysates because of their safe and efficient properties [11,29]. As shown in Table 1, defatted monkfish muscles were hydrolyzed under in vitro GI digestion, and the degree of hydrolysis (DH, %) of the resulting hydrolysate (referred to as MPTH) was 27.24% ± 1.57%, which was significantly higher than those of hydrolysates using pepsin (23.51 ± 1.96%) and trypsin (20.17% ± 1.55%), individually. Furthermore, the scavenging activities of MPTH on DPPH· and HO· at the concentration of 5.0 mg protein/mL were 44.54% ± 3.12% and 41.32% ± 2.73%, respectively, which were significantly stronger than those of hydrolysates using pepsin and trypsin, individually. Then, MPTH was chosen for the following separation process.

### 3.2. Purification of APs from MPTH 

#### 3.2.1. Fractionation of MPTH 

**Figure 1:**
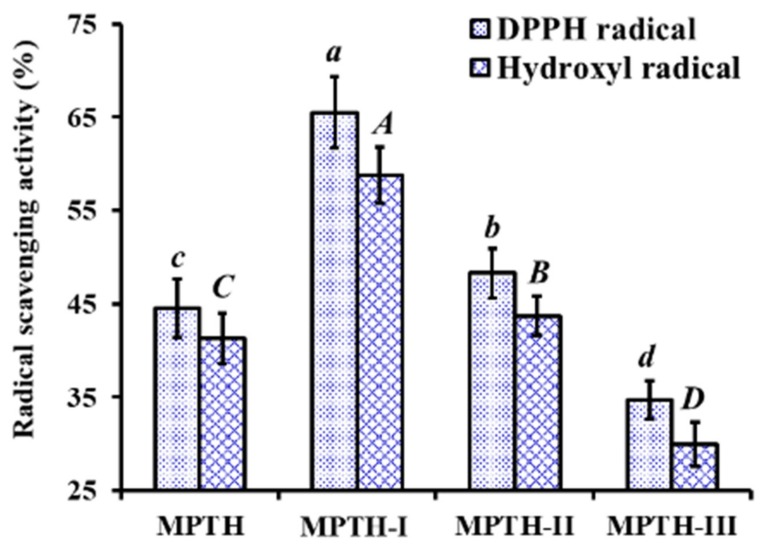
DPPH· and HO· scavenging activity of MPTH and its three fractions (MPTH-I, MPTH-II, and MPTH-III) by membrane ultrafiltration at the concentration of 5.0 mg protein/mL. The data are presented as the mean ± SD (*n* = 3). *^a–d^* or *^A–C^* column-wise values on radical scavenging activity with the same superscripts indicate no significant difference (*p* > 0.05).

Using MWCO membranes of 3 and 5 kDa, MPTH was divided into three fractions including MPTH-I (MW < 3 kDa), MPTH-II (3 kDa < MW < 5 kDa), and MPTH-III (MW > 5 kDa). At the concentration of 5.0 mg protein/mL, DPPH· and HO· scavenging activities of MPTH-I was 65.52% ± 3.86% and 58.78% ± 3.05%, respectively, which were significantly higher than those of MPTH, MPTH-II, and MPTH-III (*p* < 0.05) (Figure 1). Previous literature indicated that the antioxidant activities of protein hydrolysates were negatively related to their average MW distribution [5,30,31]. In the report, peptides in MPTH-I fraction with small MW revealed strong radical scavenging activity, which agreed with those previous reports. Therefore, MPTH-I was selected for the subsequent chromatographic separation. 

#### 3.2.2. Anion-Exchange Chromatography of MPTH-I 

**Figure 2:**
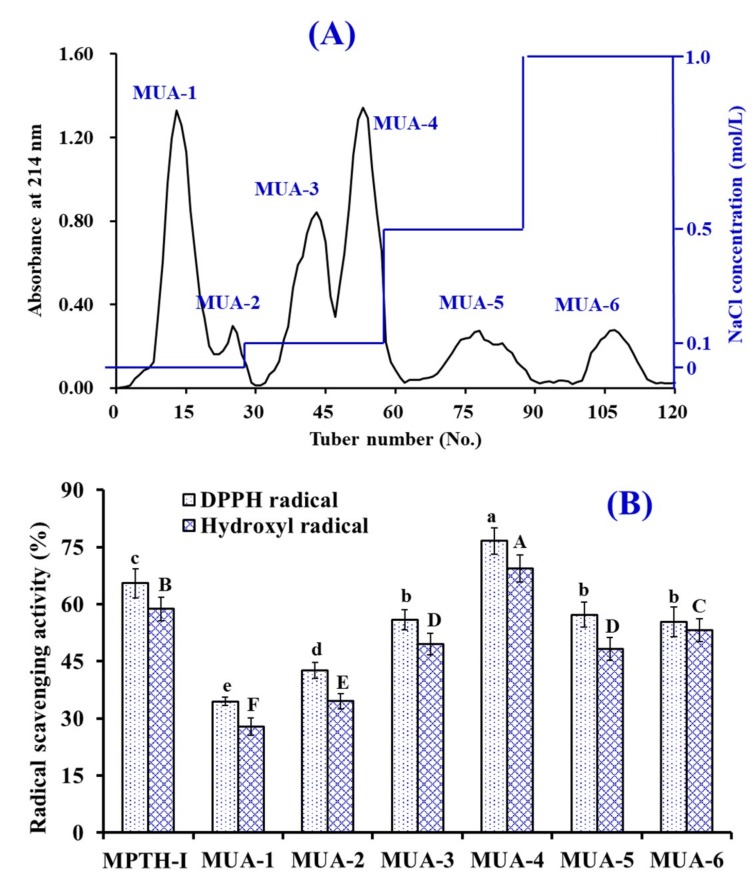
Elution profile of MPTH-I in DEAE-52 cellulose anion-exchange chromatography (**A**) and radical scavenging activity of MPTH-I and its fractions (MUA-1 to MUA-6) at the concentration of 5.0 mg protein/mL (**B**). The data are presented as the mean ± SD (*n* = 3). *^a–e^* or *^A–F^* Values with the same superscripts indicate no significant difference (*p* > 0.05).

As shown in Figure 2A, six fractions (MUA-1 to MUA-6) were separated from MPTH-I fraction using a DEAE-52 cellulose column. Amongst those fractions, MUA-1 and MUA-2 were eluted using DW, MUA-3 and MUA-4 were eluted using 0.1 M NaCl, MUA-5 was eluted using 0.5 M NaCl, and MUA-6 was eluted using 1.0 M NaCl. DPPH· and HO· scavenging activities of MPTH-I and six fractions were presented in Figure 2B, and the data made clear that DPPH· (76.64% ± 3.43%) and HO· (69.39% ± 3.58%) scavenging abilities of MUA-4 were significantly stronger than those of MPTH-I (DPPH·: 65.52% ± 3.86%; HO·: 58.78% ± 3.05%), MUA-1 (DPPH·: 34.46% ± 0.98%; HO·: 28.13% ± 2.28%), MUA-2 (DPPH·: 42.65% ± 2.16%; HO·: 34.53% ± 1.95%), MUA-3 (DPPH·: 55.91% ± 2.67%; HO·: 49.52% ± 2.83%), MUA-5 (DPPH·: 57.34% ± 3.247%; HO·: 48.25% ± 2.96%), and MUA-6 (DPPH·: 55.36% ± 3.86%; HO·: 53.22% ± 3.05%) at the concentration of 5.0 mg protein/mL (*p* < 0.05).

Acidic and hydrophobic amino acid residues in peptide sequences, such as aspartic acid (Asp), methionine (Met), glutamic acid (Glu), phenylalanine (Phe), leucine (Leu), and tyrosine (Tyr), can be adsorbed on the anion-exchange resins [5,32]. In addition, hydrophobic and acidic amino acid residues will enhance the bioactivities of peptides [21,33]. The present results suggested that the peptides in MUA-4 fraction should contain acidic and/or hydrophobic amino acid residues and those residues should be beneficial to their antioxidant activities. So, MUA-4 was selected for the following experiment.

#### 3.2.3. Gel Filtration Chromatography of MUA-4

**Figure 3:**
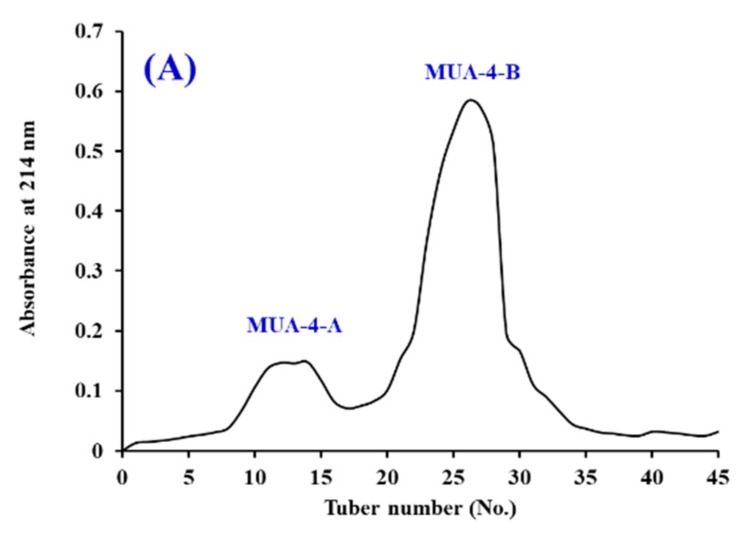

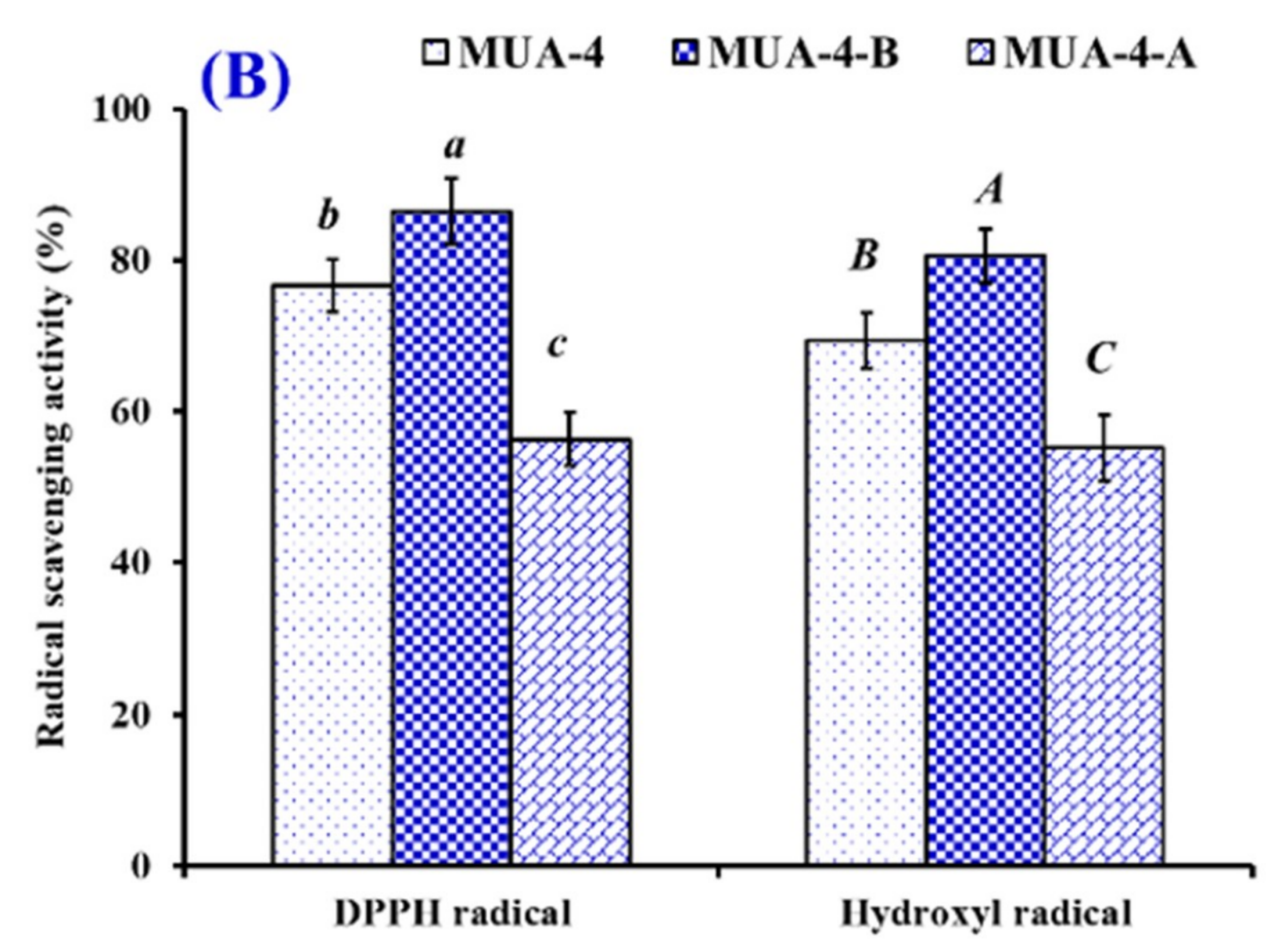
Elution profile of MUA-4 in Sephadex G-25 chromatography (**A**) and radical scavenging activities of MUA-4 and its fractions (MUA-4-A and MUA-4-B) at 5.0 mg protein/mL concentration (**B**). The data are presented as the mean ± SD (*n* = 3). *^a–c^* or *^A–C^* Column wise values with the same superscripts of this type indicate no significant difference (*p* > 0.05).

As shown in Figure 3A, MUA-4 was split into two components of MUA-4-A and MUA-4-B using a Sephadex G-25 column. Figure 3B indicated that DPPH· and HO· scavenging activities of MUA-4-B were 86.39% ± 4.32% and 80.56% ± 3.55% at the concentration of 5.0 mg protein/mL, which were significantly higher than those of MUA-4 (DPPH· 76.64% ± 3.43%; HO· 69.39% ± 3.58%) and MUA-4-A (DPPH· 56.38% ± 3.62%; HO· 55.26% ± 4.39%) (*p* < 0.05). Gel filtration chromatography is a frequently-used prepared technique according to the MW of the separated substances and is applied to separate peptides from protein hydrolysates and their fractions, such as Spanish mackerel [30], miiuy croaker [10,20], ark shell [19], and blue-spotted stingray [22]. Therefore, fraction MUA-4-B was selected for the following isolation process.

#### 3.2.4. Isolation of Peptides from MUA-4-B by RP-HPLC

**Figure 4:**
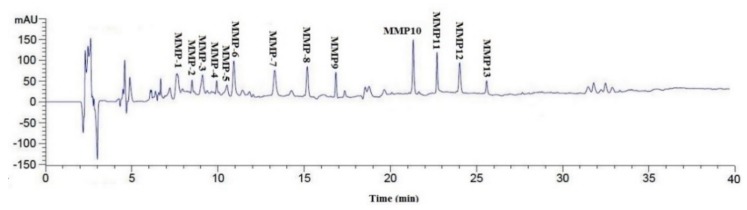
Elution profile of MUA-4-B separated by RP-HPLC system on a Zorbax, SB C-18 column (4.6 × 250 mm) from 0 to 40 min.

**Figure 5:**
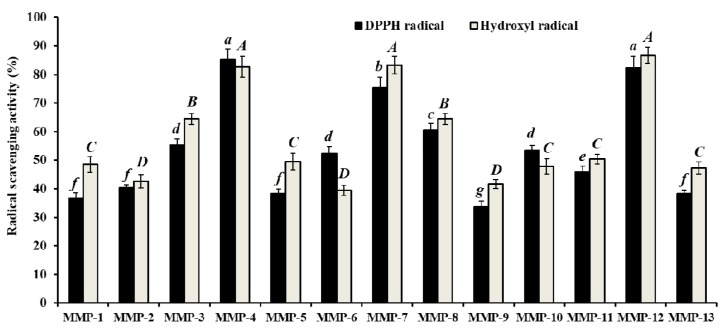
DPPH· and HO· scavenging activity of thirteen major sub-fractions (MMP-1 to MMP-13) of MUA-4-B at the concentration of 2.0 mg protein/mL. The data are presented as the mean ± SD (*n* = 3). *^a–g^* or *^A–G^* Column wise values with the same superscripts indicate no significant difference (*p* > 0.05).

RP-HPLC is an effective technique applied to purify and quantify peptides in a mixture solution on their hydrophobic character [32,34]. The retention time (RT) and the peak area can be used for qualitative and quantitative analysis of the isolated peptides [11]. As shown in Figure 4, MUA-4-B was finally purified using RP-HPLC system and thirteen major sub-fractions (MMP-1 to MMP-13) were collected separately through repeated chromatography isolation. As shown in Figure 5, thirteen fractions showed varying degrees of DPPH· and HO· scavenging activity, and the radical scavenging activities of MMP-4, MMP-7, and MMP-12 were confirmed to be higher than those of other ten peptides. Therefore, MMP-4, MMP-7, and MMP-12 with RT of 9.912 min, 13.354 min, 24.032 min, respectively, were collected and lyophilized for amino acid sequence identification and activity evaluation.

### 3.3. Amino Acid Sequence Analysis and Mass Spectrometry of APs

**Figure 6:**
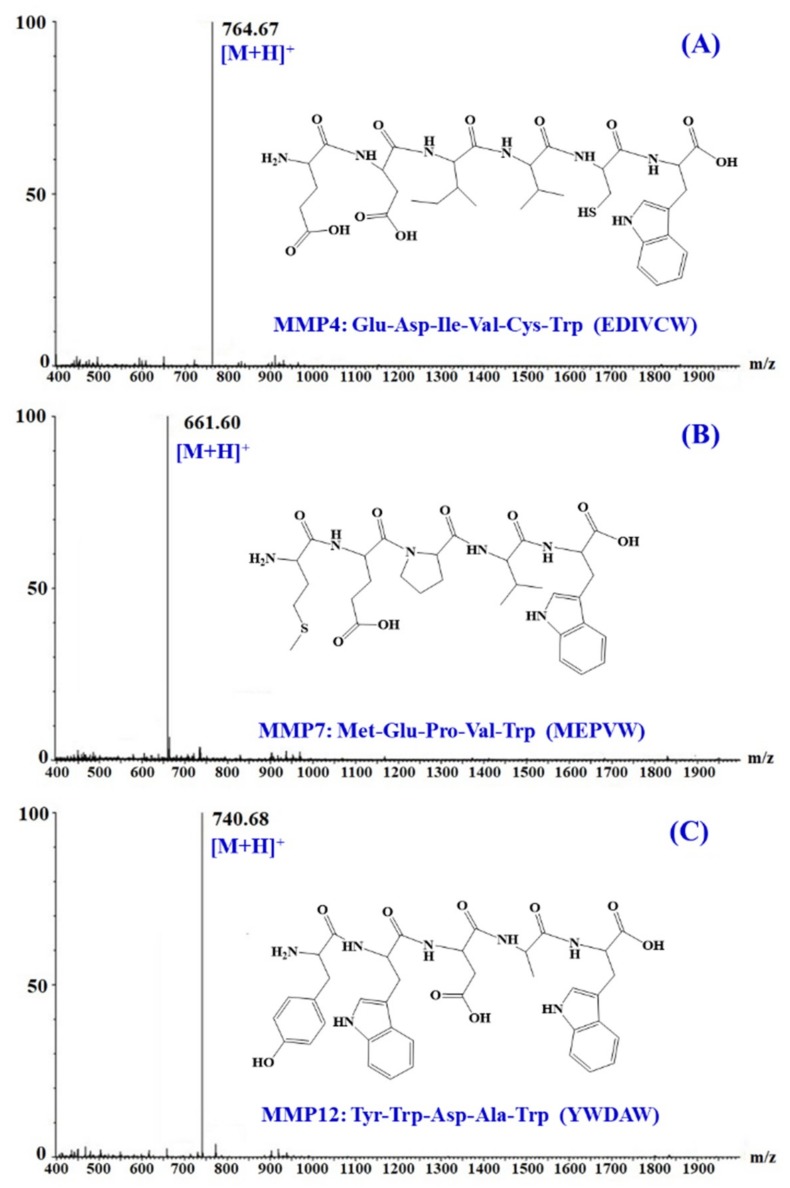
Mass spectra of three isolated peptides (MMP-4 (**A**), MMP-7 (**B**), and MMP-12 (**C**)) from protein hydrolysate of monkfish (*L. litulon*) muscle.

The amino acid sequences and molecular mass of three isolated APs (MMP-4, MMP-7, and MMP-12) were determined using protein sequencer and ESI-MS, and the results were shown in Figure 6. The amino acid sequences of three isolated peptides were identified as Glu-Asp-Ile-Val-Cys-Trp (EDIVCW, MMP-4), Met-Glu-Pro-Val-Trp (MEPVW, MMP-7), and Tyr-Trp-Asp-Ala-Trp (YWDAW, MMP-12) with MWs of 763.82, 660.75, and 739.75 Da, respectively, which were agreed well with their theoretical masses of 763.86, 660.78, and 739.77 Da.

### 3.4. Radical Scavenging Activity

#### 3.4.1. DPPH Scavenging Activity

**Figure 7:**
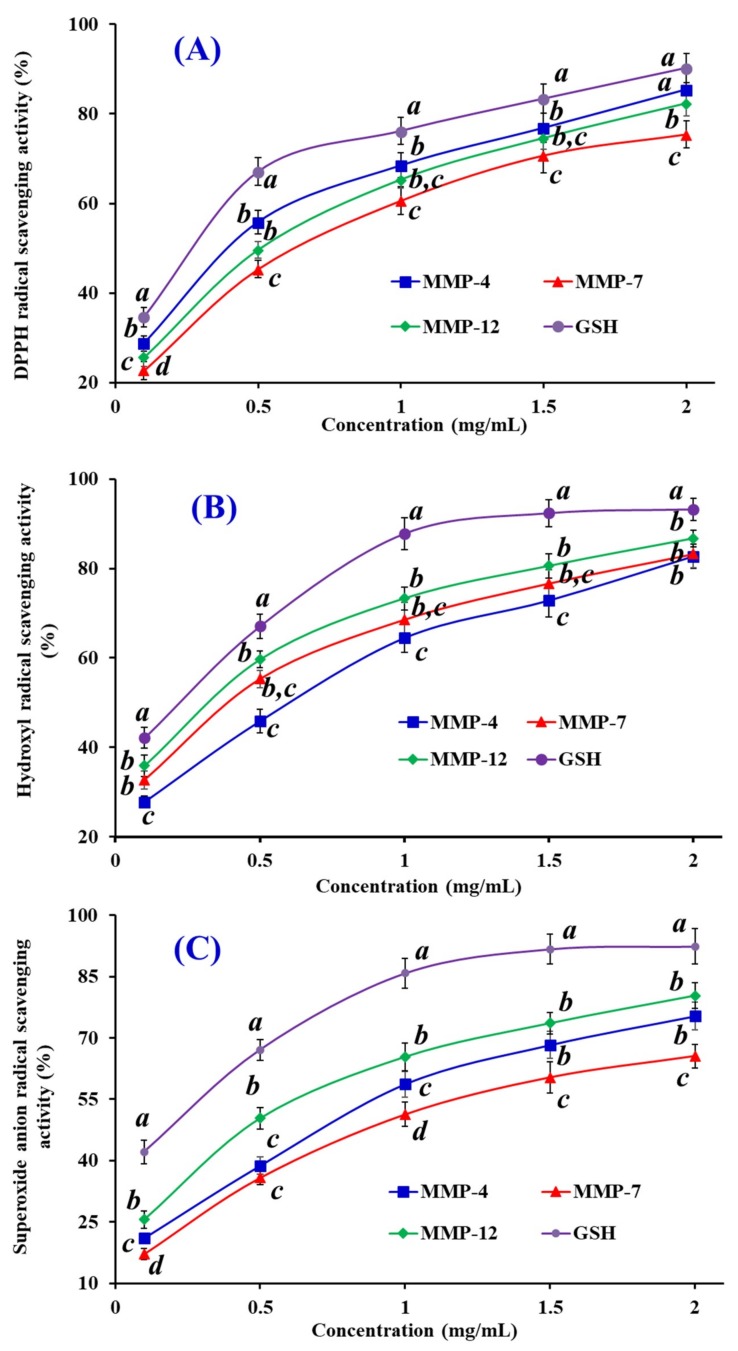
DPPH· (**A**), HO· (**B**), and $O_{2}^{-}$· (**C**) scavenging activities of three isolated peptides (MMP-4, MMP-7, and MMP-12) from protein hydrolysate of monkfish (*L. litulon*) muscle. The data are presented as the mean ± SD (*n* = 3). *^a–d^* Values with the same letters indicate no significant difference of different samples at the same concentrations (*p* > 0.05).

As shown in Figure 7A, three isolated peptides (MMP-4, MMP-7, and MMP-12) strongly scavenge DPPH· in a dose-dependent manner. The EC_50_ values of MMP-4, MMP-7, and MMP-12 were 0.39, 0.62, and 0.51 mg/mL, respectively, but their activities were still lower than that of the positive control of GSH at the same concentration. The EC_50_ value of MMP-4 was lower than those of most APs from protein hydrolysates of red stingray cartilages (VPR: 4.61 mg/mL, IEPH: 1.90 mg/mL, LEEEE: 3.69 mg/mL, and IEEEQ: 4.01 mg/mL) [6], loach (PSYV: 17.0 mg/mL) [35], salmon pectoral fin (TTANIEDRR: 2.50 mg/mL) [36], Spanish mackerel skins (PFGPD: 0.80 mg/mL, PYGAKG: 3.02 mg/mL, and YGPM: 0.72 mg/mL) [37], skipjack tuna bone (GADIVA: 0.57 mg/mL) [23], and miiuy croaker swim bladders (FPYLRH: 0.51 mg/mL; GIEWA: 0.78 mg/mL; YLPYA: 3.63 mg/mL; VPDDD: 2.87 mg/mL) [20]. Therefore, the present result suggested that three isolated peptides (MMP-4, MMP-7, and MMP-12), especially MMP-4 could contribute an electron or hydrogen radical to suppress the DPPH· reaction.

#### 3.4.2. HO· Scavenging Activity

The abilities of three isolated peptides (MMP-4, MMP-7, and MMP-12) on scavenging HO· were investigated and the data presented in Figure 7B. MMP-4, MMP-7, and MMP-12 with the EC_50_ values of 0.61, 0.38, and 0.32 mg/mL, respectively, could concentration-dependently affect their radical activities. The EC_50_ value of MMP-12 was less than those of APs from weatherfish loach (PSYV: 2.64 mg/mL) [38], Spanish mackerel skins (PFGPD: 0.81 mg/mL, PYGAKG: 0.66 mg/mL, and YGPM: 0.88 mg/mL) [37], grass carp skin (PYSFK: 2.28 mg/mL; VGGRP: 2.06 mg/mL) [39], red stingray cartilages (VPR: 0.77 mg/mL, IEPH: 0.46 mg/mL, LEEEE: 0.70 mg/mL, and IEEEQ: 1.30 mg/mL) [6], bluefin leatherjacket heads (GPP: 2.385 mg/mL; WEGPK: 5.567 mg/mL; GVPLT: 4.149 mg/mL) [32], and miiuy croaker swim bladders (FPYLRH: 0.68 mg/mL; GIEWA: 0.71 mg/mL; YLPYA: 2.90 mg/mL; VPDDD: 2.85 mg/mL) [20]. Excess HO· in organisms during oxidative stress can destroy cell membranes and key biomacromolecules, which further cause a series of chronic diseases [5,16]. As the efficient APs, three isolated peptides (MMP-4, MMP-7, and MMP-12) can act as HO· scavengers for significantly alleviating the oxidative damage in biological systems.

#### 3.4.3. $O_{2}^{-}$· Scavenging Assay

Figure 7C indicated the $O_{2}^{-}$· scavenging rates of three isolated peptides (MMP-4, MMP-7, and MMP-12) obviously increased when their concentrations increased from 0.1 to 2.0 mg/mL, but their scavenging abilities were weaker than that of GSH at the same concentration. EC_50_ values of MMP-4, MMP-7, and MMP-12 were 0.76, 0.94, and 0.48 mg/mL, respectively. The EC_50_ value of MMP-12 was lower than those of MMP-4, MMP-7, and other APs from croceine croaker muscle (VLYEE: 0.69 mg/mL; MILMR: 0.99 mg/mL) [40], skipjack tuna bone (GADIVA: 0.52 mg/mL) [23], Spanish mackerel skins (PFGPD: 0.91 mg/mL, PYGAKG: 0.80 mg/mL, and YGPM: 0.73 mg/mL) [37], bluefin leatherjacket heads (WEGPK: 3.22 mg/mL; GPP: 4.67 mg/mL; GVPLT: 2.88 mg/mL) [32], and miiuy croaker swim bladders (YLPYA: 3.61 mg/mL; VPDDD:4.11 mg/mL) [20]. However, the EC_50_ value of MMP-12 was higher than those of FPYLRH (0.34 mg/mL) and GIEWA (0.30 mg/mL) from miiuy croaker swim bladders [20]. $O_{2}^{-}$· is the most common free radical generated in vivo, and can promote oxidative reaction to generate peroxy and HO·. Superoxide dismutase (SOD) protects the cell from the deleterious effects of $O_{2}^{-}$· in living organisms. Therefore, MMP-4, MMP-7, and MMP-12 can be applied to eliminate $O_{2}^{-}$· damage together with SOD in biological systems.

### 3.5. Lipid Peroxidation Inhibition Assay

**Figure 8:**
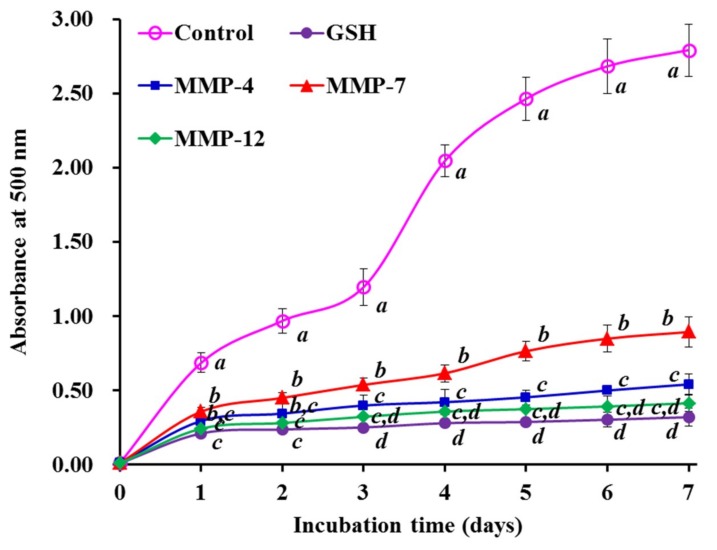
Lipid peroxidation inhibition assays of three isolated peptides (MMP-4, MMP-7, and MMP-12) from protein hydrolysate of monkfish (*L. litulon*) muscle. The data are presented as the mean ± SD (*n* = 3). *^a–d^* Values with the same letters indicate no significant difference of different samples at the same concentrations (*p* > 0.05).

Figure 8 showed the inhibiting abilities of three isolated peptides (MMP-4, MMP-7, and MMP-12) on the lipid peroxidation system in the linoleic acid model. The absorbance at 500 nm of three isolated peptide solutions was significantly lower than that of the negative control (without antioxidant). Moreover, MMP-4 and MMP-12 showed similar abilities on lipid oxidation inhibition to that of GSH.

The oxidative process is drawn into multifarious reactions for lipid hydroperoxides and radical propagation when oxygen is present in organism or food products, but the radical scavenging assays only measured an antioxidant property, which cannot reflect its role as an antioxidant to protect organism and/or food systems from lipid oxidation [11,40]. Consequently, lipid peroxidation inhibition assay is popular to evaluate the comprehensive antioxidant capacity of antioxidant molecules [2]. The presented results suggested that MMP-4 and MMP-12 could effectively delay and control lipid peroxidation during 7 days incubation in the tested system. In addition, the flaw that the activity of MMP-4 and MMP-12 is weaker than that of artificial antioxidants can be compensated by increasing their using dose.

### 3.6. Protective Activity of MMP-4, MMP-7, and MMP-12) on H_2_O_2_-induced Oxidative Damage in HepG2 Cells

#### 3.6.1. Cytotoxicity of MMP-4, MMP-7 and MMP-12 on HepG2 Cells

**Figure 9:**
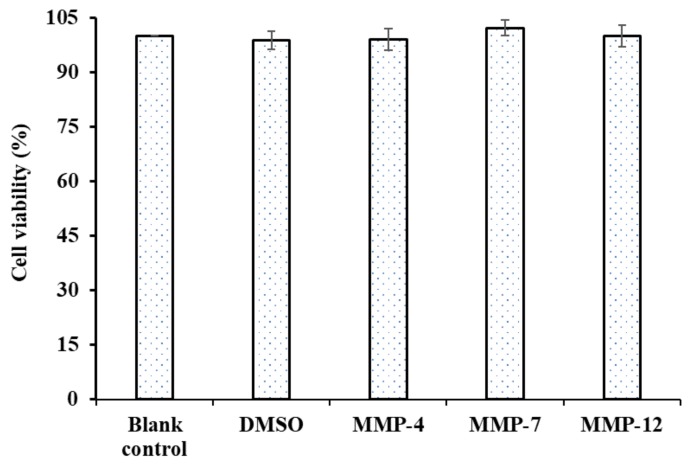
Cytotoxicity of three isolated peptides (MMP-4, MMP-7, and MMP-12) from protein hydrolysate of monkfish (*L. litulon*) muscle on HepG2 cells at the concentration of 100.0 µM. The data are presented as the mean ± SD (*n* = 3).

Cytotoxic effects of three isolated peptides (MMP-4, MMP-7, and MMP-12) at the concentrations of 100.0 µM were determined in HepG2 cells by the MTT assay. As shown in Figure 9, MMP-4, MMP-7, and MMP-12 exhibited no significant cytotoxic effects on HepG2 cells compared to the blank control (no peptide treatment) at the tested concentrations for 24 h treatment (*p* < 0.05). The result indicated that three isolated peptides (MMP-4, MMP-7, and MMP-12) from protein hydrolysate of monkfish muscle could serve as the candidate molecules for antioxidant food and drugs.

#### 3.6.2. Protection of MMP-4, MMP-7, and MMP-12 on H_2_O_2_-induced Oxidative Damage HepG2 Cells

Oxidative stress caused by ROS is responsible for a wide variety of cellular damage and is the most validated mechanism of secondary injury. Following oxidative stress, the overproduction of ROS and subsequently the depletion of antioxidants results in the total breakdown of the endogenous antioxidant defense mechanisms, culminates in failure to protect cells from damage induced by oxidative stress, and finally leads to significantly reduced cell viability which we try to protect with antioxidant peptides. In the present study, we showed that H_2_O_2_ could damage HepG2 cells and caused cells significantly cytotoxic effects. In our previous report, H_2_O_2_ at the concentration of 300 μM is used to establish the oxidative stress model of HepG2 cells in the assay [2], and the cell viability reached 48.85 ± 1.68% of control group for induced 24 h. Using the oxidative stress model of HepG2 cells, the cytoprotective effects of MMP-4, MMP-7, and MMP-12 were evaluated through measured the cell viability, ROS content, activities of antioxidant enzymes and levels of MDA.

**Figure 10:**
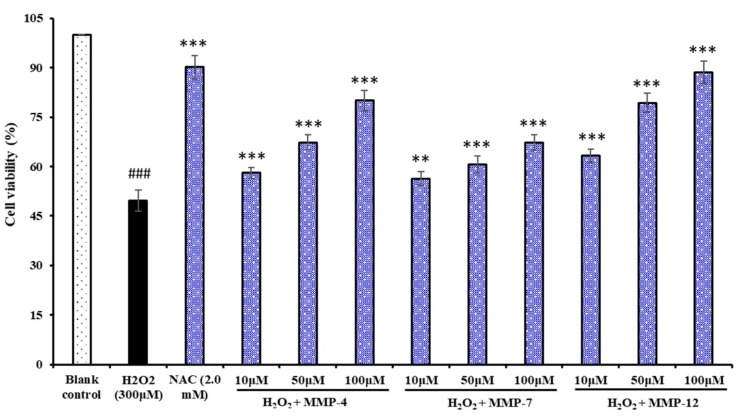
Protective effects of three isolated peptides (MMP-4, MMP-7, and MMP-12) on H_2_O_2_-induced oxidative damage in HepG2 cells at concentrations of 10.0, 50.0, and 100.0 100.0 µM. N-Acetylcysteine (NAC) was used as the positive control. The data are presented as the mean ± SD (*n* = 3). ^###^ *p* < 0.001 versus the blank control group. ** *p* < 0.01 versus the H_2_O_2_ treated group. *** *p* < 0.001 versus the H_2_O_2_ treated group.

Figure 10 showed the influences of three isolated peptides (MMP-4, MMP-7, and MMP-12) on H_2_O_2_-induced oxidative damage HepG2 cells, and the cell viability of three isolated peptides (MMP-4, MMP-7, and MMP-12) treated groups was gradually increased in a concentration-dependent manner compared with the model group. Moreover, the MMP-12 treated group increased the HepG2 cell viability from 48.85% ± 1.68% to 63.28% ± 2.06%, 79.35% ± 2.85%, and 88.65% ± 3.42% at the concentrations of 10.0, 50.0, and 100.0 μM, respectively, which were significantly higher than that of the model group (*p* < 0.01). The finding suggested that MMP-4, MMP-7, and MMP-12 could strongly protect H_2_O_2_-induced oxidative damage HepG2 cells, especially at the high concentrations.

#### 3.6.3. Effect of MMP-4, MMP-7, and MMP-12 on the Levels of ROS in Oxidative Damage HepG2 Cells

**Figure 11:**
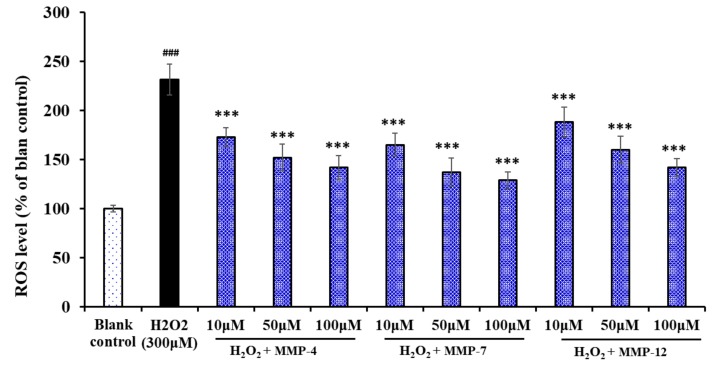
Effects of MMP-4, MMP-7, and MMP-12 on the levels of ROS in oxidative damage HepG2 cells at the concentration of 10, 50, and 100 μM. The data are presented as the mean ± SD (*n* = 3). ^###^ *p* < 0.001 *versus* the blank control group; *** *p* < 0.001 *versus* the H_2_O_2_ treated group.

The effects of MMP-4, MMP-7, and MMP-12 on the levels of ROS in oxidative damage HepG2 cells were measured and shown in Figure 11, the level of ROS observed in the HepG2 exposed to H_2_O_2_ was 231.7% ± 13.5% of the blank control, which was extremely (*p* < 0.001) significantly higher than those of the blank control group. As expected, the intracellular ROS levels were significantly attenuated by MMP-4, MMP-7, and MMP-12 pretreatment in a dose-effect manner. Among them, MMP-7 showed the strongest scavenging effect of ROS and decreased the ROS level from 229.5% ± 16.8% to 165.2% ± 11.9%, 137.3% ± 14.3%, and 129.1% ± 8.6% at the concentrations of 10, 50, and 100 μM. ROS are generated by different physiological and biochemical oxidative processes in the organisms and associated with numerous physiological and pathophysiological processes [41,42]. At high levels, ROS damage some key biological macromolecules and play a major role in the pathogenesis of various human diseases [43,44]. The present data indicated that MMP-4, MMP-7, and MMP-12 could protect cells from the damage of ROS.

#### 3.6.4. Effects of MMP-4, MMP-7, and MMP-12 on the Antioxidant Enzymes and MDA in Oxidative Damage HepG2 Cells

**Figure 12:**
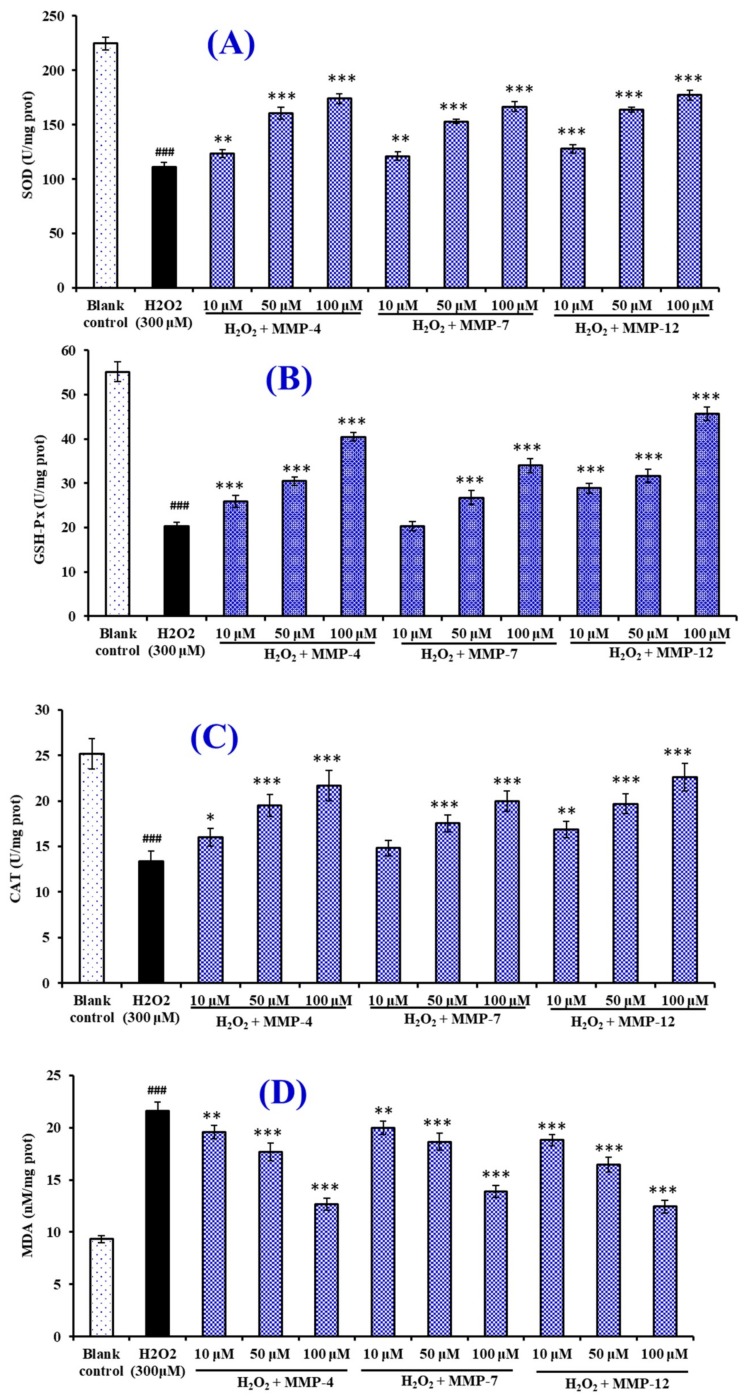
The effects of MMP-4, MMP-7, and MMP-12 on the levels of intracellular antioxidant enzymes (SOD (**A**), CAT (**B**), and GSH-Px (**C**)) and content of MDA (**D**) in H_2_O_2_-induced HepG2 cells. All data are presented as the mean ± SD (*n* =3 ). ^###^ *p* < 0.001 *versus* the control group; * *p* < 0.05, ** *p* < 0.01, and *** *p* < 0.001 *versus* the H_2_O_2_ treated group.

Figure 12A–C showed the effects of MMP-4, MMP-7, and MMP-12 on the levels of intracellular antioxidant enzymes (SOD, CAT, and GSH-Px). Compared with the normal control group, the levels of SOD, CAT, and GSH-Px were significantly decreased by H_2_O_2_ in the HepG2 cells, which indicated that the oxidative stress badly damaged the intracellular antioxidant enzymes of HepG2. It is exciting that the SOD, CAT, and GSH-Px activities of HepG2 cells incubated by MMP-4, MMP-7, and MMP-12 increased in a dose-dependent manner and were significantly higher than the H_2_O_2_ damaged group (*p* < 0.05). In addition, the HepG2 cell groups incubated by MMP-12 showed the highest levels of SOD, CAT, and GSH-Px at the same tested concentrations, and followed by the group incubated by MMP-4. Figure 12D indicated that the MDA content (21.63 ± 0.81 nM/mg prot) of HepG2 cells in the damage group was significantly (*p* < 0.05) increased compared with that of the control group (9.32 ± 0.35 nM/mg prot). At the concentration of 10.0, 50.0, and 100 μM, the MDA content of MMP-12 incubated group was 18.8 ± 0.56, 16.46 ± 0.74, and 12.43 ± 0.62 nM/mg prot, which was lower than those of MMP-4, MMP-7, and the H_2_O_2_ treated groups. Therefore, MMP-4, MMP-7, and MMP-12 could reduce the oxidative stress injury by the restoration of endogenous antioxidation and the decrease of lipid peroxidation.

Intracellular antioxidant enzymes, such as SOD, CAT, and GSH-Px, are the most important factors for preserving cellular and systemic health through holding back the radicals attacking cellular membrane and components. MDA is the oxidative metabolite of cell lipid oxidation, and the reduction of oxidative damage in cells was accompanied by the decrease of oxidative metabolites, which give an index to the degree of oxidative damage in cells. Nazeer et al. reported that KTFCGRH from croaker (*Otolithes ruber*) muscle protein hydrolysate could increase an endurance capacity and facilitate recovery from oxidative stress because the peptide could efficiently quench radicals, inhibit the lipid peroxidation and DNA damage, and improve the activities of endogenous cellular antioxidant enzymes (CAT, GST and SOD) [45]. You et al. reported that loach peptide (LP) can increase an endurance capacity and facilitate recovery from fatigue of mice because LP can increase the levels of blood glucose and liver glycogen, decrease the levels of lactic acid and blood urea nitrogen, and improve the activities of endogenous cellular antioxidant enzymes (SOD, CAT, and GSH-Px) [35]. The present results indicated that three isolated peptides (MMP-4, MMP-7, and MMP-12) have similar activity to protect H_2_O_2_-induced HepG2 cells from oxidative stress by enhancing endogenous antioxidant enzyme defense system.

### 3.7. Structure-Activity Relationship of MMP-4, MMP-7, and MMP-12

The structure-activity relationship of the functional molecule will provide the basis for predicting the activity of new compounds, but so far it is still difficult to illuminate the relationship between structural characteristics and antioxidant activity of peptides [5,46]. In general, molecular size, hydrophobicity, and amino acid composition and sequence are regarded as key factors for the activity of antioxidant peptides [47,48].

Low-MW antioxidant peptides have a highly pronounced ability to hold back the oxidative stress because they can easily interact with target radicals to terminate the free radical chain reactions [5,49,50]. In addition, Li et al. reported that the antioxidant activities of protein hydrolysates with average MW (AMW) from 0.64 kDa to 257.19 kDa were negatively related to the logarithm of their AMW [31]. The MWs of MMP-4, MMP-7, and MMP-12 were 763.82, 660.75, and 739.75 Da, which help them easily interacting with ROS to prevent oxidative stress.

The hydrophobic and aromatic amino acids are believed to play an important role in the antioxidant activity of peptides [5,51]. The abundance of hydrophobic amino acids, such as Pro, Met, Leu, Tyr, Phe and Val in the peptide sequences seems to enhance the antioxidant activities by increasing peptide solubility in lipids, and the interaction with radical species [52,53,54]. Zheng et al. reported that hydrophobic amino acid residues (Leu and Ile) in AEDKKLIQ played an important role on the hydrophobic targets [55]. Met residue acted as a reactive site in the PMRGGGGYHY, WMFDW, WMGPY, and EMGPA through formatting a sulfoxide structure to scavenge free radicals [18,56]. Pro residue can help antioxidant peptides, such as LDEPDPL, WMGPY and EMGPA, scavenge ROS because of the pyrrolidine ring [18,57]. Furthermore, aromatic groups of Trp, Phe, and Tyr residues can scavenge free radicals by providing protons [55,56,57,58]. Therefore, hydrophobic/aromatic amino acid residues in the sequences of EDIVCW (Ile, Val, Cys and Trp), MEPVW (Met, Pro, Val and Trp), and YWDAW (Tyr, Trp, Ala and Trp) play an important role for their antioxidant activity.

Polar amino acid residues, especially acidic amino acid residues, play a crucial part in the HO· scavenging and metal ion chelating capacities of antioxidant peptides [57,59]. The same result was reported by Zheng et al. that Glu, Asp and Lys within the sequence of AEDKKLIQ were conducive to the radical scavenging potential and metal-chelating activity [55]. In addition, the antioxidant function of Asp seemed to have nothing to do with where it appears in the sequence of peptides [55]. Therefore, acid amino acids, including the Glu and Asp residues in DIVCW, and Glu residue in MEPVW, should be very helpful in improving their antioxidant ability.

Interestingly, recent reports showed that melatonin could protect human erythrocytes from H_2_O_2_-induced oxidative damage on anion exchange capability through Band 3 in the absence of the endogenous erythrocytes antioxidant system [60]. Therefore, we will also look at whether three isolated peptides (MMP-4, MMP-7, and MMP-12) have similar functions to melatonin.

## 4. Conclusions

In the experiment, thirteen antioxidant peptides (MMP-1 to MMP-13) were isolated from the hydrolysate of monkfish (*Lophius litulon*) muscle prepared using in vitro gastrointestinal (GI) digestion and three peptides (MMP-4, MMP-7, and MMP-12) were identified as EDIVCW, MEPVW, and YWDAW, respectively, which exhibited high in vitro antioxidant activities through radical scavenging, reducing power, and lipid peroxidation inhibition assays. More importantly, EDIVCW, MEPVW, and YWDAW could protect H_2_O_2_-induced HepG2 cells from oxidative stress by decreasing ROS and MDA levels and enhancing endogenous antioxidant enzyme (SOD, CAT, and GSH-Px) defense system, which demonstrated that pre-treatment with the antioxidant peptides led to the protection of hepatic cells against H_2_O_2_-induced cell death through a mechanism that involves decreasing ROS and MDA levels and enhancing endogenous antioxidant enzyme defense system. These present results provided a scientific basis for the hepatoprotective effects of antioxidant peptides, especially YWDAW, derived from the protein hydrolysate of monkfish and suggested that YWDAW may be of therapeutic value in the treatment of some liver diseases associated with oxidative stress. In addition, animal feeding experiments on the three isolated peptides (MMP-4, MMP-7, and MMP-12) will be conducted to evaluate their in vivo antioxidant effects and structure-function relationship with or without endogenous antioxidant systems in our lab.

## Figures and Tables

**Table 1:** Degree of hydrolysis (%) and radical scavenging activity (%) of the protein hydrolysate of monkfish muscle using pepsin, trypsin, and in vitro gastrointestinal (GI) digestion model.

Protease	Degree of Hydrolysis (%)	Radical Scavenging Activity (5.0 mg Protein/mL, %)	
		DPPH·	HO·
Pepsin	23.51 ± 1.96 ^b^	33.24 ± 2.34 ^b^	29.34 ± 1.48 ^b^
Trypsin	20.17 ± 1.55 ^c^	29.21 ± 1.87 ^c^	27.74 ± 2.15 ^c^
In vitro GI digestion	27.24 ± 1.57 ^a^	44.54 ± 3.12 ^a^	41.32 ± 2.73 ^a^

The data are presented as the mean ± SD (*n* = 3). ^a–c^ Values with the same letters in each column indicate no significant difference (*p* > 0.05).
